# *Bacillus subtilis* and *Pichia farinose* mixture improves growth performance and nutrient absorption capacity in broiler chicks

**DOI:** 10.3389/fvets.2023.1086349

**Published:** 2023-03-23

**Authors:** Huan Wang, Lu Fu, Jian Ying Zhang, In Ho Kim

**Affiliations:** ^1^School of Biology and Food Engineering, Chuzhou University, Chuzhou, China; ^2^Department of Animal Resources Science, Dankook University, Cheonan, Republic of Korea; ^3^China Light Industry Hesheng Technology Co., Ltd, Chuzhou, China; ^4^College of Life Science, Linyi University, Linyi, China

**Keywords:** *Pichia farinose*, *Bacillus subtilis*, probiotic mixture, broiler chick, nutrient absorption

## Abstract

**Introduction:**

This study evaluated the effects of dietary supplementation of Bacillus subtilis and Pichia farinose mixture (BPM) on growth performance, apparent ileal digestibility, cecal bacteria counts, small intestinal morphology and digestive enzymes activities, and jejunal nutrient transporters gene expression in broiler chicks.

**Methods:**

A total of 768 one-day-old Ross 308 broiler chicks were randomly Q18 assigned into 3 groups based on the initial body weight (42.00 ± 0.08 g). The experimental periods were 35 days. There were 16 replicates per group and 16 birds per cage. Dietary treatments included a basal diet supplemented with 0, 0.1, or 0.2% BPM to form CON, BPM0.1 (consisting Bacillus subtilis with 1.0 × 107 viable spore and Pichia farinose with 1.0 × 107 viable spore per kg diet), and BPM0.2 (consisting Bacillus subtilis with 2.0 × 107 viable spore and Pichia farinose with 2.0 × 107 viable spore per kg diet) groups.

**Results and discussion:**

Dietary supplementation of graded levels of BPM has positive effects on growth performance of broiler chicks, manifesting in the increase of body weight gain during days 1–35 as well as the decrease of feed conversion ratio during days 1–7, 21–35, and 1–35. Moreover, BPM supplementation positively improved ileal energy and crude protein digestibility, increased Lactobacillus counts, optimized intestinal morphology, enhanced intestinal digestive enzymes activities, and upregulated jejunal SGLT-1, GLUT-2, and PEPT-1 expression. Therefore, BPM supplementation improved growth performance of broiler chicks, which was partially related to the improvement in intestinal nutrient absorption capacity.

## Introduction

Antibiotics have been widely used in poultry husbandry to improve their growth performance and feed efficiency for a long time. However, the abuse of antibiotics has been proved to lead to antibiotic resistance in microbial communities. People are trying to find alternatives to antibiotics in poultry husbandry (1). Probiotics have great potential to be used as a substitute for antibiotics in the diet of broiler chicks to improve their growth performance (2–4). They stimulate the development of beneficial microbiota and disturb the colonization of pathogens in the intestine, and further regulate gastrointestinal functions and enhance enteric immune system (5, 6).

Multiple probiotic strains such as *Lactobacillus, Pediococcus, Bifidobacterium*, and *Bacillus* spp., have been recorded to be used in the diet of animals and humans (7–10). The *Bacillus* spp. has been reported to be able to regulate the intestinal environment by producing extracellular enzymes, and further enhancing the digestibility and absorption of nutrients in the gut (11, 12). The *Pichia* spp. may also have the same action mode (10). Despite the benefits of *Bacillus* and *Pichia* spp. have been observed in the application with single-strain. However, no study has investigated the effects of *Bacillus* and *Pichia* spp. mixture on the production performance in broiler chicks. It is reported that multiple-strain probiotics appear to be more effective in improving the growth performance and health status of animals than single probiotic strains (13). The application of multiple-strain probiotics provides such thoughts as more strains imply a broader spectrum of efficacy, even additive and/or synergistic effects. Based on the results observed in our previous study, we found that feeding finishing pigs with *Bacillus subtilis* and *Pichia farinose* mixture (BPM) containing diet have positive effects on their growth performance and intestinal microbiota (13).

Therefore, we hypothesized that dietary supplementation of BPM positively improved growth performance of broiler chicks, which was achieved by regulating intestinal beneficial bacteria counts and improving intestinal nutrient absorption capacity. The objective of this study was to evaluate the effects of dietary supplementation of BPM on growth performance, apparent ileal digestibility, cecal bacteria counts, small intestinal morphology and digestive enzymes activities, and jejunal nutrient transporters gene expression in broiler chicks.

## Materials and methods

The experimental protocol describing the management and care of animals were revised and approved by the Animal Care and Use Committee of Dankook University, Cheonen, South Korea.

### Source of probiotic complex

The probiotic complex (BioPro-alpha) was obtained from a commercial company (Deaho, Seoul, Korea). This product contained a spray-dried mixture of *Bacillus subtilis* (1.0 × 10^7^ viable spore/g of product) and *Pichia farinose* (1.0 × 10^7^ viable spore/g of product) in the form of powder (http://www.daeho.com/eng/index.html; Supplementary material 1).

### Experimental design, animals, and diets

A total of 768 one-day-old mixed-gender Ross 308 broiler chicks were purchased from a commercial hatchery (Yang Ji Company, Cheonan, South Korea) and raised in stainless steel cages (1.75 × 1.55 m) and fed experimental feed for 35 days. The house was provided with programmable lighting and ventilation. Feed and water were provided *ad libitum* and diets were prepared in pellet form. Broiler chicks were raised in a room (32°C) for the first 3 days and the temperature reduced by 2°C every week until 24°C maintaining humidity around 65%. Birds were randomly assigned to 3 groups based on the initial body weight (42.00 ± 0.08 g). There were 16 replicates per group and 16 birds per cage. Dietary conditions were based on a basal diet supplemented with 0, 0.1, or 0.2 % BPM to form CON, BPM0.1 (consisting *Bacillus subtilis* with 1.0 × 10^7^ viable spore and *Pichia farinose* with 1.0 × 10^7^ viable spore per kg diet), and BPM0.2 (consisting *Bacillus subtilis* with 2.0 × 10^7^ viable spore and *Pichia farinose* with 2.0 × 10^7^ viable spore per kg diet) groups. Experimental periods were divided into 3 phases (phase 1, 1–7; phase 2, 8–21; phase 3, 22–35). Diets (Table 1) were formulated according to the recommendation of NRC (14).

**Table 1:** Composition and nutrient levels of the experimental basal diet, (%, as-fed basis).

	**Feeding phases**		
**Items**	**Starter (days 1–7)**	**Grower (days 7–21)**	**Finisher (days 21–35)**
**Ingredients (%)**			
Corn	53.41	55.09	58.59
Soybean meal	25.70	22.60	19.61
Wheat bran	0.30	0.30	0.30
Wheat flour	5.00	5.00	5.00
Rubber seed meal	-	2.00	-
Canola	-	2.00	-
Corn gluten meal	2.90	-	-
Sesame meal	2.00	2.00	2.00
Corn distiller's dried grains with solubles	3.00	3.00	5.00
Meat meal	2.00	3.00	3.00
Tallow	1.00	1.80	3.10
Soy oil	0.50	-	-
Limestone	1.33	1.25	1.29
Dicalcium phosphate	0.77	0.19	0.35
Salt	0.33	0.26	0.24
Methionine (99%)	0.36	0.33	0.34
Lysine HCl	0.83	0.63	0.67
Threonine	0.19	0.18	0.14
Choline chloride (60%)	0.13	0.10	0.10
Vitamin-Mineral premix^a^	0.20	0.20	0.20
Phytase	0.05	0.07	0.07
Total	100	100	100
**Analyzed composition, %**			
Crude protein	21.99	20.49	18.49
Crude fat	4.08	4.95	6.08
Crude fiber	2.44	2.66	2.40
Crude ash	5.85	5.27	5.06
Metabolizable energy, kcal/kg	3,045	3,135	3,251
Available phosphorus	0.45	0.43	0.42
Calcium	0.96	0.90	0.89
Lysine	2.20	1.68	1.52
Methionine	0.97	0.91	0.76
Tryptophan	0.26	0.23	0.21
Methionine + cysteine	1.05	0.99	0.93

^a^Provided per kilogram of complete diet: vitamin A, 10 000 IU; vitamin D_3_, 2000 IU; vitamin E, 48 IU; vitamin K_3_, 1.5 mg; riboflavin, 6 mg; niacin, 40 mg; d-pantothenic, 17 mg; biotin, 0.2 mg; folic acid, 2 mg; choline, 166 mg; vitamin B_6_, 2 mg; and vitamin B_12_, 28 mg, Fe (as FeSO_4_•7H_2_O), 90 mg; Cu (as CuSO_4_•5H_2_O), 15 mg; Zn (as ZnSO_4_), 50 mg; Mn (as MnO_2_), 54 mg; I (as KI), 0.99 mg; and Se (as Na_2_SeO_3_•5H_2_O), 0.25 mg.

### Sampling and measurements

#### Feed analysis

Diets were analyzed for crude protein (CP), calcium (Ca), phosphorus (P), and amino acids (AA) by AOAC (15) procedures. CP was determined by the Kjeldahl methodology (N × 6.25). Dietary Ca was analyzed by atomic absorption spectrophotometry after wet ash procedures, and P was determined by colorimetric procedure. For the determination of methionine (Met) and cysteine (Cys), the samples were oxidized with performic acid overnight at 0°C. Met and Cys composition was measured using an AA Analyzer (Beckman 6300, Beckman Coulter, Inc., Fullerton, CA) after 24-h hydrolysis in HCl.

#### Growth performance

Broiler chicks were weighed pen-basis to calculate the body weight gain (BWG). Feed intake (FI) was recorded on days 1, 7, 21, and 35. The information was then used to calculate FCR.

#### Apparent ileal digestibility

During days 28-35, feeding broiler chicks with 0.2% chromium oxide containing diet. On the final day, a portion of the small intestine from Meckel's diverticulum proximal to the ileocecal junction was removed in order to collect ileal digesta samples for apparent ileal digestibility (AID) measurements. According to the procedure established by the AOAC (15), the crude protein (nitrogen × 6.25; method 968.06) and dry matter (method 930.15) composition in the digesta samples were analyzed. The combustion heat was measured by a bomb calorimeter (Parr 6100; Parr Instrument Co., Moline, IL, USA) to determine the gross energy content of the feed and excreta samples. Chromium concentrations were determined by atomic absorption spectrophotometry (UV-1201, Shimadzu, Kyoto, Japan). The apparent digestibility values for ileal nutrients were calculated by a formula provided by Dang et al. (16).

#### Cecal bacteria counts

The digesta contents of the birds were collected from the caeca into micro-tubes. Samples were placed on ice and transported to the laboratory, where analysis was immediately carried out. One g of cecal digesta sample was blended with 9 ml sterile peptone water and mixed for 1 min on a vortex stirrer. Counts of viable bacteria in the cecal samples were determined by plating serial 10-fold dilutions (10^−3^ to 10^−6^) onto MRS agar (Difco Laboratories, Detroit, MI, USA) and MacConkey agar (Difco Laboratories, Detroit, MI, USA) plates to isolate *Lactobacillus* and *Escherichia coli*, respectively. The MRS agar plates were then incubated for 24 h at 37°C under anaerobic conditions. The MacConkey plates were incubated for 24 h at 37°C under aerobic conditions. After the incubation periods, colonies of the respective bacteria were counted and expressed as the logarithm of colony-forming units per g (log_10_ CFU/g).

#### Small intestinal samples preparation

The whole small intestine was removed and the adherent material of the small intestine was carefully removed under ice-cold saline, weighed, and separated into the duodenum, jejunum, and ileum. About the 1-cm long segment from the middle of intestinal part were taken in duplicate and placed in 2 separate tubes. One sample was fixed with 10% neutral-buffered formalin solution for histology and the other sample was frozen in liquid nitrogen, and then stored at −80°C for measuring digestive enzymes activities and nutrient transporter gene expression.

#### Small intestinal morphology

The preparation of the intestinal sample cutting block was the same as the above. A microtome was used to make five cuts that were 5 μm. The cuts were stained with hematoxylin-eosin. The values were measured using a light microscope. Measurements of villus height and depth were determined at a magnification of 10X. A minimum of five measurements per slide were made for each parameter and averaged into one value (17). Values presented means from 10 adjacent villi and only vertically oriented villi were measured.

#### Intestinal digestive enzymes activities

To extract the broiler digestive enzymes and quantify the protein concentration, the segment of duodenum, jejunum, and ileum were homogenized in 0.2 M phosphate buffer pH 7 (1:5 w/v), using a microhomogenizer (THP-220; Omni International, Kennesaw, GA, USA). The homogenate was centrifuged at 13,000 × g, at 4°C for 20 min, and aliquots prepared which were kept at −20°C until use. The protein concentration from a crude enzyme extract was compared to a standard curve of bovine serum albumin (BSA), within a linear range, according to the standard method of Lowry et al. (18).

#### Jejunal nutrient transporter gene expression

Total RNA was isolated from muscle samples using RNAiso Reagent (TaKaRa, Dalian, Liaoning, China). The RNA integrity was assessed by electrophoresis on a 1% agarose gel containing formaldehyde. The RNA concentration was measured using a Beckman DU-640 spectrophotometer (Beckman). The sequences of primers for the genes tested were specifically designed according to the sequences located in GenBank (Table 2). The total RNA samples were purified and subjected to reverse transcription using the Takara PrimeScript RT Reagent Kit with gDNA Eraser (Takara, Dalian, China) and processed for cDNA synthesis as per Takara PrimeScript RT instructions (19). The relative expression levels of sodium/glucose cotransporter protein-1 (SGLT-1), glucose transporter-2 (GLUT-2), and peptide transporter 1 (PEPT-1) genes in pectoral muscle were analyzed by RT-PCR, which was performed in a 10 μL reaction mix containing 1 μL 2 × SYBR Premix Ex Taq II (TakaRa, Dalian, China), 3 μL dH2O, 0.5 μL of the upstream and downstream primers, and 1 μL cDNA using a Bio-Rad CFX-96 thermocycler (Bio-Rad, CA). The reaction conditions were as follows: initial denaturation at 95°C for 30 s and 44 cycles of amplification at 72°C for 30 s. The annealing was carried out for 40 s at temperatures specific to each target gene. At the end of the amplification, step-wise melting curves were performed to confirm the product specificity. The cytoskeletal protein, β-actin, was used as the internal reference. The gene expression levels of the samples were determined by the 2^−ΔΔCt^ method (20).

**Table 2:** Primers used for quantitative real-time PCR.

**Gene**	**Product (bp)^a^**	**Primer sequences**	
β-Actin	158	Forward	GCCCAGCACGATGAAGAT
		Reverse	ATTTACGGTGGACGATGGAC
PEPT-1	205	Forward	CCCCTGAGGAGGATCACTGTT
		Reverse	CAAAAGAGCAGCAGCAACGA
SGLT-1	126	Forward	GTAACATTGGCAGCGGACAT
		Reverse	TGGGTACAAACAGCCATCCT
GLUT-2	118	Forward	CAGTTCTTCCTGCTCCTGCT
		Reverse	TCATCGGGTCACAGTTTCCT

SGLT-1, sodium/glucose cotransporter protein-1; PEPT-1, peptide transporter 1; GLUT2, glucose transporter-2.

^a^PCR product size (base pairs).

### Statistical analysis

All data were statistically analyzed using the General Linear Model procedure (SAS Inst. Inc., Cary, NC, USA) in a randomized completely block design. The replicate cage was used as the experimental unit. Orthogonal contrasts were used to examine the linear and quadratic effects in response to the increase of dietary BPM concentrations. Variability in the data was expressed as the standard error of means (SEM), *P* < 0.05 was considered statistically significant.

## Results

Dietary supplementation of graded levels of BPM linearly increased BWG during days 1-35 (*P* = 0.031), whereas linearly decreased FCR during days 1-7 (*P* = 0.008), 21-35 (*P* = 0.016), and 1–35 (*P* = 0.001). However, BPM supplementation did not affect FI (Table 3).

**Table 3:** Effect of dietary supplementation of *Bacillus subtilis* and *Pichia farinose* mixture (BPM) on growth performance of broiler chicks^*^.

	**BPM, %**			**SEM**	***P*** **-value**		
**Items**	**0.0**	**0.1**	**0.2**		**ANOVA**	**Linear**	**Quadratic**
Initial body weight, g	42.08	41.95	41.98	0.105	0.672	0.532	0.528
**BWG, g**							
Days 1–7	146.5	149.5	152.2	2.746	0.356	0.153	0.971
Days 7–21	645.6	648.5	652.7	8.907	0.853	0.576	0.956
Days 21–35	987.1	1000	1015	10.93	0.200	0.074	0.957
Days 1–35	1779	1798	1820	13.00	0.095	0.031	0.940
**FI, g**							
Days 1–7	160.1	156.3	158.2	2.759	0.621	0.626	0.400
Days 7–21	1004	996.6	987.7	6.599	0.205	0.077	0.950
Days 21–35	1730	1722	1718	9.732	0.675	0.387	0.863
Days 1–35	2895	2875	2864	13.18	0.255	0.105	0.785
**FCR**							
Days 1–7	1.09	1.05	1.04	0.014	0.017	0.008	0.264
Days 7–21	1.56	1.54	1.52	0.023	0.427	0.196	0.917
Days 21–35	1.76	1.73	1.70	0.017	0.052	0.016	0.999
Days 1–35	1.63	1.60	1.57	0.011	0.004	0.001	0.912

BWG, body weight gain; FI, feed intake; FCR, feed conversion ratio; SEM, standard error of the mean.

^*^Values represent the means of 16 replicate cages (*n* = 16) per treatment.

Energy (*P* = 0.027) and crude protein (*P* = 0.025) digestibility increased linearly with the dose of BPM increased in the diet, but dry matter digestibility did not differ among groups (Table 4).

**Table 4:** Effect of dietary supplementation of *Bacillus subtilis* and *Pichia farinose* mixture (BPM) on apparent ileal digestibility of broiler chicks^*^.

	**BPM, %**			**SEM**	***P*** **-value**		
**Items, %**	**0.0**	**0.1**	**0.2**		**ANOVA**	**Linear**	**Quadratic**
Dry matter	83.48	85.125	85.44	0.708	0.134	0.064	0.449
Gross energy	84.49	86.08	86.80	0.689	0.075	0.027	0.615
Crude protein	78.48	81.35	81.41	0.858	0.038	0.025	0.195

SEM, standard error of the mean.

^*^Values represent the means of 16 replicate cages with 3 birds per replicate cage (*n* = 16) per treatment.

Broiler chicks fed with BPM containing diet led to an increase in cecal *Lactobacillus* counts (*P* = 0.025) in a dose-dependent manner, but cecal *Escherichia coli* did not affect by BPM supplementation (Table 5).

**Table 5:** Effect of dietary supplementation of *Bacillus subtilis* and *Pichia farinose* mixture (BPM) on cecal bacteria counts of broiler chicks^*^.

	**BPM, %**			**SEM**	***P*** **-value**		
**Items**	**0.0**	**0.1**	**0.2**		**ANOVA**	**Linear**	**Quadratic**
*Lactobacillus*, log_10_ cfu g^−1^	7.62	7.69	7.74	0.038	0.074	0.025	0.794
*Escherichia coli*, log_10_ cfu g^−1^	6.25	6.19	6.16	0.037	0.273	0.122	0.676

SEM, standard error of the mean.

^*^Values represent the means of 16 replicate cages with three birds per replicate cage (*n* = 16) per treatment.

Effects of graded levels of BPM supplementation on small intestinal morphology of broiler chicks were shown in Table 6. As observed, the villus height and its ratio to crypt depth in duodenum and ileum were increased linearly with the dose of BPM increased in the diet (*P* < 0.05). Additionally, linear decreases in jejunal and ileal crypt depth were observed in broiler chicks fed with graded levels of BPM-containing diet (*P* < 0.05).

**Table 6:** Effect of dietary supplementation of *Bacillus subtilis* and *Pichia farinose* mixture (BPM) on duodenal, jejunal, and ileal morphology of broiler chicks^*^.

	**BPM, %**			**SEM**	***P*** **-value**		
**Items**	**0.0**	**0.1**	**0.2**		**ANOVA**	**Linear**	**Quadratic**
**Duodenum**							
Villus height, μm	1,181	1,194	1,211	9.366	0.097	0.033	0.879
Crypt depth, μm	133.8	133.2	132.7	1.065	0.755	0.459	0.965
Villus height to crypt depth ratio	8.83	8.97	9.13	0.083	0.057	0.018	0.907
**Jejunum**							
Villus height, μm	808.6	810.6	815.5	7.796	0.813	0.537	0.877
Crypt depth, μm	140.9	138.9	137.6	0.834	0.034	0.011	0.756
Villus height to crypt depth ratio	5.74	5.84	5.93	0.073	0.220	0.085	0.993
**Ileum**							
Villus height, μm	515.0	524.7	538.5	6.604	0.061	0.020	0.804
Crypt depth, μm	144.8	139.8	138.2	1.237	0.003	0.001	0.261
Villus height to crypt depth ratio	3.56	3.76	3.90	0.058	0.002	0.001	0.685

SEM, standard error of the mean.

^*^Values represent the means of 16 replicate cages with 3 birds per replicate cage (*n* = 16) per treatment.

Lipase activity in duodenum (*P* = 0.027), amylase activity in jejunum (*P* = 0.002), cellulase activity in jejunum (*P* = 0.018), and cellulase activity in ileum (*P* = 0.003) increased linearly with the dose of BPM increased in the diet (Table 7).

**Table 7:** Effect of dietary supplementation of *Bacillus subtilis* and *Pichia farinose* mixture (BPM) on duodenal, jejunal, and ileal digestive enzymes activities of broiler chicks^*^.

	**BPM, %**			**SEM**	***P*** **-value**		
**Items**	**0.0**	**0.1**	**0.2**		**ANOVA**	**Linear**	**Quadratic**
**Duodenum**							
Trypsin, mU/mg protein	4.47	4.59	4.68	0.227	0.802	0.512	0.963
Amylase, mU/mg protein	2,157	2,243	2,313	58.77	0.196	0.075	0.908
Cellulase, mU/mg protein	412.9	413.9	414.1	7.696	0.992	0.908	0.969
Lipase, mU/mg protein	123.5	126.7	132.1	2.393	0.134	0.027	0.350
**Jejunum**							
Trypsin, mU/mg protein	4.12	4.12	4.18	0.108	0.898	0.676	0.852
Amylase, mU/mg protein	2,243	2,306	2,491	48.32	0.004	0.002	0.314
Cellulase, mU/mg protein	417.2	426.7	436.5	5.325	0.056	0.018	0.983
Lipase, mU/mg protein	143.4	148.9	142.9	3.674	0.456	0.927	0.217
**Ileum**							
Trypsin, mU/mg protein	3.79	3.89	3.94	0.097	0.530	0.274	0.931
Amylase, mU/mg protein	1,952	1,958	1,971	32.82	0.913	0.682	0.917
Cellulase, mU/mg protein	463.0	492.5	493.8	6.524	0.004	0.003	0.091
Lipase, mU/mg protein	151.9	153.1	157.2	2.838	0.399	0.202	0.670

Abbreviation: SEM, standard error of the mean.

^*^Values represent the means of 16 replicate cages with 3 birds per replicate cage (*n* = 16) per treatment.

Moreover, we observed that the expression of SGLT-1 (*P* = 0.005), GLUT-2 (*P* = 0.012), and PEPT-1 (*P* = 0.009) in jejunum increased linearly with the dose of BPM increased in the diet (Table 8).

**Table 8:** Effect of dietary supplementation of *Bacillus subtilis* and *Pichia farinose* mixture (BPM) on jejunal nutrient transporter gene expression of broiler chicks^*^.

	**BPM, %**			**SEM**	***P*** **-value**		
**Items**	**0.0**	**0.1**	**0.2**		**ANOVA**	**Linear**	**Quadratic**
SGLT-1	1.00	1.19	1.24	0.053	0.011	0.005	0.299
GLUT-2	1.00	1.18	1.26	0.068	0.037	0.012	0.604
PEPT-1	1.00	1.21	1.38	0.093	0.031	0.009	0.828

SGLT-1, sodium/glucose cotransporter protein-1; GLUT-2, glucose transporter-2; PEPT-1, peptide transporter 1; SEM, standard error of the mean.

^*^Values represent the means of 16 replicate cages with 3 birds per replicate cage (*n* = 16) per treatment.

## Discussion

Some studies have reported that dietary supplementation of probiotics is able to improve the growth performance of broiler chicks (21). In the present study, feeding broiler chicks with graded levels of BPM containing positively improved their growth performance. Tarabees et al. (22) reported that broiler chicks fed the diet supplemented with probiotic mixture improved the BWG and decreased the FCR. Fazelnia et al. (23) also observed an increase in BWG and a decrease in FCR caused by broiler chicks fed with probiotic mixture containing diet. Therefore, the BWG of broiler chicks increased by BPM supplementation was considered to be related to the reduction of FCR, which corresponded to the improvement of nutrient digestibility (24–26).

As expected, in this study, broiler chicks fed the diet supplemented with BPM linearly increased AID of crude protein and energy. Similarly, Zhang and Kim (27) noted that dietary supplementation of multi-strain probiotics decreased FCR and increased the AID of amino acids in broiler chicks. Giang et al. (28) observed an increase of apparent ileal crude protein, crude fiber, and organic matter digestibility as well as a decrease of FCR in broiler chicks consuming probiotic complexes containing diet. We considered that the growth performance positively affected by BPM supplementation was partially related to the reduction of FCR caused by nutrient digestibility improvement.

Microbiota presents in the intestine play a key role in improving feed efficiency and/or nutrient digestibility (29). The supplementation of probiotics is a suitable strategy to regulate intestinal microbiota. Probiotics are able to inhibit the colonization of pathogenic bacteria in the intestine (30). Tarabees et al. (22) reported that broiler chicks fed the diet supplemented with probiotic mixture increased the counts of beneficial bacteria while decreased the counts of harmful bacteria in the cecum. In the present study, we also observed an increase in cecal *Lactobacillus* counts caused by feeding broiler chicks with graded levels of BPM containing diet. Recently, a gut metagenomic analysis study in broiler chicks revealed the prominent roles of cecal *Lactobacillus* counts in the improvement of feed efficiency in broiler chicks. They reported that the feed efficiency was related to the abundance of *Lactobacillus* in the cecum (31). We considered that the supplementation of BPM was beneficial to regulate the intestinal microbiota, thus generate positive effects on the growth performance.

Additionally, the development of the gastrointestinal tract is critical to optimize the growth performance of poultry in the early growth stage (32). Digestion and absorption of nutrients occur in the small intestine of birds (33, 34). In the small intestine, abundant villi endow a large surface area for nutrient absorption and enzyme secretion (35). In general, the villus height, crypt depth, and their ratio are common parameters to be used to reflect the small intestine development (36). Giannenas et al. (37) reported that the supplementation of probiotics stimulated the development of villus height in broiler chicks. Sen et al. (38) demonstrated that feeding broiler chicks with probiotics containing diet increased villus height and the ratio of villus height to crypt depth in both duodenum and ileum in a dose-dependent manner. In the present study, we also observed that BPM supplementation improved intestinal morphology. We speculated that the supplementation of BPM was beneficial to enhance the nutrient absorption capacity of broiler chicks by modulating intestinal morphology.

Moreover, the digestive enzymes play an important role in digesting nutrients into smaller nutritional molecules so as to facilitate the absorption by the host (39). Abdel-Moneim et al. (40) noted that the activities of small intestinal proteases, lipase, and amylase increased in Japanese quail fed with probiotic containing diet. Gong et al. (41) also observed that the activities of trypsin, amylase, lipase, and protease in duodenum increased in feeding broiler chicks with probiotic containing diet. We also observed an increase in small intestinal digestive enzyme activities in the current study. We considered that the BPM supplementation was beneficial to enhance digestive enzyme activities, and therefore improved nutrient digestibility.

The transport mediators expressed in the apical and basal membranes of enterocytes are important to achieve nutrient digestion and absorption (42, 43). Nutrients are transported into enterocytes by special transporters (44). Glucose is the key fuel and important metabolic substrate in poultry, it is absorbed by intestinal epithelium via the apically located SGLT-1 and transported into the blood via GLUT-2 which is expressed on the basolateral membrane (45, 46). PEPT-1 is a solute carrier for oligopeptides. It acts as a cotransporter in intestine by a proton-dependent manner (47). In the present study, dietary supplementation of BPM upregulated the expression of SGLT-1, GLUT-2, and PEPT-1 in the jejunum. Similarly, Faseleh Jahromi et al. (48) reported that feeding broiler chicks with probiotic mixture containing diet had pronounced effects on the expression of GLUT-2 and SGLT-1 in the small intestine. Wang et al. (49) also observed the nutrient transporter genes of laying hens upregulated, and the production performance further improved, by probiotic supplementation. Therefore, the supplementation of BPM was beneficial to enhance the nutrient absorption capacity.

## Conclusion

In this study, feeding broiler chicks with BPM containing improved growth performance of broiler chicks, which was partially attributed to the improvement in nutrient absorption capacity, manifesting in the increase of cecal beneficial bacteria counts, optimization of intestinal morphology, enhancement of intestinal digestive enzyme activities, and the upregulation of nutrient transporter gene expression.

## Data availability statement

The raw data supporting the conclusions of this article will be made available by the authors, without undue reservation.

## Ethics statement

The animal study was reviewed and approved by the Animal Care and Use Committee of Dankook University, Cheonen, South Korea.

## Author contributions

HW: writing—original draft, investigation, and writing—review and editing. LF and JZ: formal analysis and investigation. IK: conceptualization, methodology, supervision, and writing—review and editing. All authors contributed to the article and approved the submitted version.
