# Supplementary material for: Bacillus subtilis and Pichia farinose mixture improves growth performance and nutrient absorption capacity in broiler chicks
Source: Front Vet Sci. 2023 Mar 23;10:1086349. doi: 10.3389/fvets.2023.1086349 (PMC10076679; doi:10.3389/fvets.2023.1086349)
Supplement: Supplementary file 1 [file Data_Sheet_1.pdf]

Probiotics

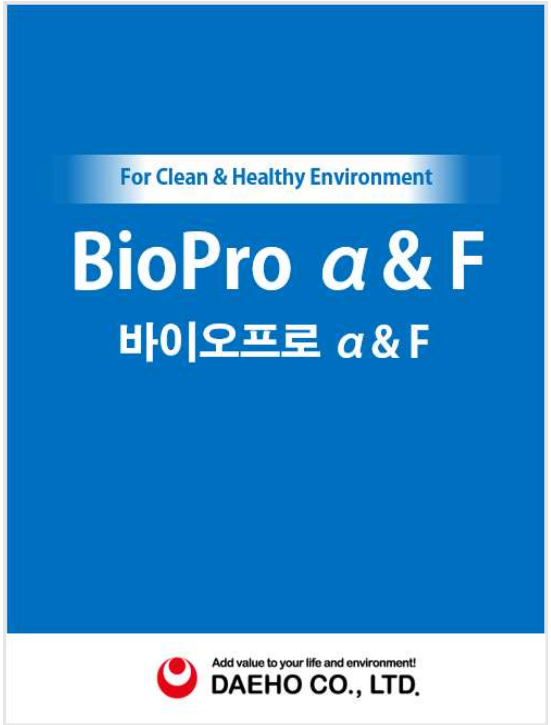

BioPro α & F

**Species**	Swine, Poultry, Cow
**Shape**	Powder(BioPro α) Liquid(BioPro F)
**Packing unit**	5kg - OPP / 25kg - Paper bag
**Ingredient/content**	BioPro α<br>-Bacillus subtilis<br>-Pichia farinosa<br><br>BioPro F<br>-Bacillus subtilis<br>-Pichia farinosa<br>+ Pine cone powder (stench masking effect)

Features | Efficacy | Usage / Amount | Etc

Features

2) Solid fermentation with high metabolites

3) Composed of abundant beneficial bacteria and metabolite

4) High stability with its acid resistance, bile resistance, and heat resistance

DAEHO CO.,LTD. **CEO**: Hyung Ho, Lee **Business Registration Number** : 124-81-34275
Head Office and Factory : 24, Jeongmunsongsan-ro 241beon-gil, Yanggam-myeon, Hwaseong-si, Gyeonggi-do, Korea T. 82-31-352-4083 F. 82-31-352-4084
Seoul Office : 40, Dongjak-daero, Seocho-gu, Seoul, Republic of Korea T. 82-2-596-2001~5 F. 82-2-596-2006

Copyrightⓒ 2017 DAEHO CO.,LTD. All Rights Reseved
